# Supplementary material for: A functional approach towards the design, development, and test of an affordable dynamic prosthetic foot
Source: PLoS One. 2022 May 6;17(5):e0266656. doi: 10.1371/journal.pone.0266656 (PMC9075626; doi:10.1371/journal.pone.0266656)
Supplement: S2 Dataset — This dataset presents the cost summary and results of the characterization tests obtained performed on the LFT ankle materials, composite blade materials and foam materials. (DOCX) [file pone.0266656.s002.docx]

# Cost summary


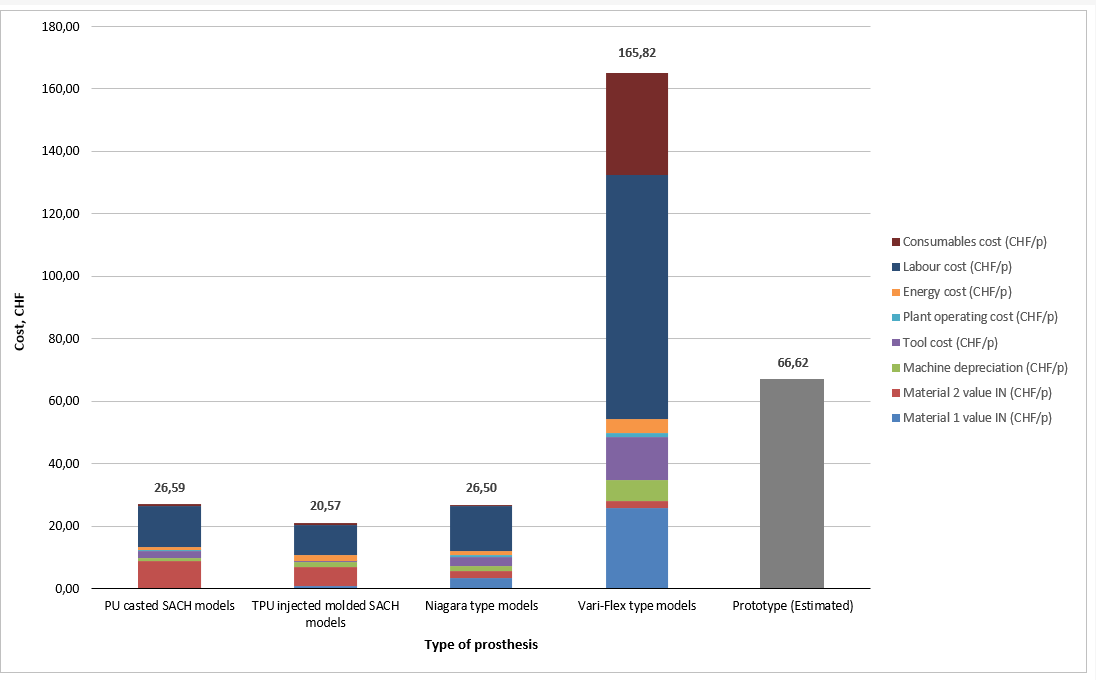


Figure 1 Cost comparison summary using cost modelling process

# Ankle material testing summary

Different LFT materials were investigated during this work. LFT injection molded samples (thickness from 3.8 to 4 mm, width 10 mm and length 85- 100 mm) were tested in a 3 point bending mode at 10 Hz and 16:1 span length: thickness ratio using a Bose hydraulic testing machine.

Table 1 Ankle testing materials fatigue testing summary

| **No** | **Material** | **Type** | **Fibre weight, %** | **σtest , MPa** | **Break at, Cycles** | **Stopped at, Cycles** |
| --- | --- | --- | --- | --- | --- | --- |
| 1 | Carbon | Supplier 1 | 30 | 175 | - | 3'000’000 |
| 2 |  |  | 40 | 175 | 142'000 | - |
|  |  |  |  | 162.5 | 4'835'201 | - |
|  |  |  |  | 125 | - | 4'179'651 |
| 3 | Glass |  | 60 | 125 | 2'300 | - |
| 4 | Carbon | Supplier 2 | 30 | 200 | 4'001 | - |
|  |  |  |  | 175 | 655'201 | - |
|  |  |  |  | 162.5 | - | 7'782'651 |
|  |  |  |  | 150 | - | 6'048'251 |
|  |  |  |  | 125 | - | 3'096'551 |
| 5 |  |  | 40 | 225 | 362'000 | - |
|  |  |  |  | 212.5 | - | 7'000'000 |
|  |  |  |  | 200 | - | 5'000'000 |
|  |  |  |  | 175 | - | 6'000'000 |
| 6 | Glass |  | 60 | 175 | 43'380 | - |
|  |  |  |  | 162.5 | 811'094 | - |
|  |  |  |  | 150 | - | 5'000'000 |
| 7 |  |  |  | 150 | 236'351 | - |
|  |  |  |  | 125 | 1'693'201 | - |

# Blade material testing summary

Several mechanical tests were conducted on blade composite samples manufactured at the LPAC laboratory under industrial conditions. All fatigue tests were done at 5Hz in 3 point bending mode using a Bose hydraulic testing machine. The samples were cut width of 10 mm and with a span length 16:1. A summary of the results are provided in Table 2.

Table 2 Blade testing materials fatigue testing summary

| **No.** | **Material** | **Type** | **Special Comments** | **Cycle time, hr** | **Process** | **Void Content, %** | **Target Stress, MPa** | **Fatigue test results** |
| --- | --- | --- | --- | --- | --- | --- | --- | --- |
| 1 | UD 300GSM | Carbon Epoxy UD | +45/-45/0_10_/+45/-45  Avg thickness: 4mm | 3  (1 hr dwell) | Autoclave at 5 bars with vacuum @ 120 °C | 0.7 | 746 | Passed – 795 MPa tested 855 MPa failed |
| 2 |  |  | +45/-45/0_9_/-45/+45  Avg thickness: 4mm | 3  (1 hr dwell) | Autoclave at 3 bars with vacuum @ 120 °C | 0.35 +-0.1 |  | Passed - 735 MPa tested  830 MPa failed |
| 3 |  |  | +45/-45/0_9_/-45/+45  Avg thickness: 4mm | 3  (1 hr dwell) | OOA with vacuum only @ 120 °C | 2.35 +-1.1 |  | Passed – 782 MPa tested Failed 897 MPa  (Quality sensitive) |
| 4 | UD 310GSM | Carbon Epoxy UD | +45/-45/0_9_/-45/+45  Avg thickness: 4mm | 3 hours  (1 hr dwell) | Autoclave with 3 bars with vacuum @ 130 °C | 0.32 | 800 | 717 MPa failed with delamination in skin |
| 5 |  |  | +45/-45/0_9_/-45/+45  Avg thickness: 4mm | 1.5 hour  (cold in cold out ) | Press with 3 bars @ 135 °C | 0.25 |  | Passed –767 MPa tested -854 MPa failed with skin delamination |
| 6 |  |  | +45/-45/0_9_/-45/+45  Avg thickness: 4mm | 20 mins  (hot in hot out ) | Press with 3 bars @ 155 °C | 0.39 |  | 725 MPa failed with delamination in skin |

# Foam material testing summary

Different foams as shown in Figure 2 were tested under cycling compression loads using a Bose hydraulic testing machine. The sample dimensions were 13 mm diameter and 10 mm height and tested at 2Hz and 70% deformation. The final selected foam composition for this work is mentioned as 55_8_foot_1 in the graph.


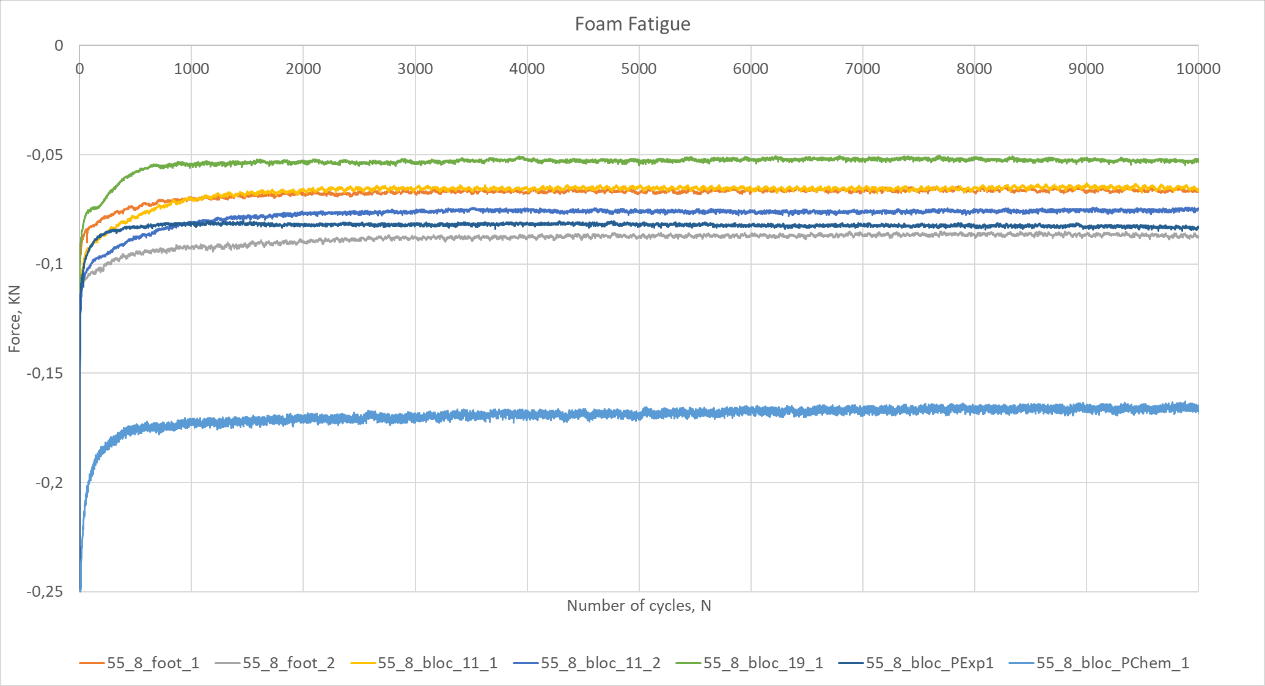


Figure 2 Fatigue test results on different foam samples
