# Supplementary material for: A functional approach towards the design, development, and test of an affordable dynamic prosthetic foot
Source: PLoS One. 2022 May 6;17(5):e0266656. doi: 10.1371/journal.pone.0266656 (PMC9075626; doi:10.1371/journal.pone.0266656)
Supplement: S1 Appendix — (PDF) [file pone.0266656.s005.pdf]

Agilis – VIETCOT measurements

Feedback questionnaire

**Subject**	
**Date**	
**Prosthesis**	
**Duration (days)**	

(1) : Stability while standing

TERRIBLE EXCELLENT

(2) : Stability while walking

TERRIBLE EXCELLENT

(3) : Stability during stance

TERRIBLE EXCELLENT

(4) : Possibility of squatting

TERRIBLE EXCELLENT

(5) : Powerful push-off

TERRIBLE EXCELLENT

(6) : Rolling-off in a supple way

TERRIBLE EXCELLENT

(7) : Feeling of firm contact with the ground

TERRIBLE EXCELLENT

(8) : Ability to turn on the prosthetic leg

TERRIBLE EXCELLENT

(9) : Ability to walk quickly

TERRIBLE EXCELLENT

(10) : Ability to climb staircase

TERRIBLE EXCELLENT

(11) : Fatigue during walking

EXHAUSTED NO FATIGUE

(12) : Rate the weight of the prosthetic foot

TERRIBLE EXCELLENT

(13) : Rate the comfort of the prosthetic foot

TERRIBLE EXCELLENT

(14) Overall grade to the prosthesis

TERRIBLE EXCELLENT

(15) : Specific comments on the foot regions

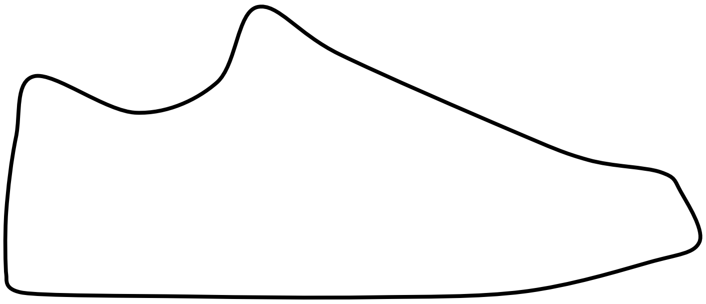

(16) : General comments on the prosthesis
