# Supplementary material for: A functional approach towards the design, development, and test of an affordable dynamic prosthetic foot
Source: PLoS One. 2022 May 6;17(5):e0266656. doi: 10.1371/journal.pone.0266656 (PMC9075626; doi:10.1371/journal.pone.0266656)
Supplement: Attachment — Submitted filename: reviewers_comments_main.docx [file pone.0266656.s007.docx]

# Reviewer #1

This paper and work aim to address an important gap in prosthetic practice in LMICs by designing a new dynamic foot. The work involves a number of design and testing stages, as would be typically needed for the development of prosthetic componentry. The design process is in itself complex, and not all facets should be considered as research. While I can appreciate the work that has gone into developing the foot, the novelty is not entirely clear. Moreover, the paper appears to lack adequate rigour and details in many facets of the work as detailed below.

### Abstract:

While it may be true that the prototype foot "showed a better performance and acceptance by users compared to a SACH foot", did it perform adequately well to be deemed a dynamic foot as was the goal? The paper needs to clearly present this here, and in the discussion section.

*The assessment of the K3-Level requires additional testing by a certified institution. We cannot assert that our foot fulfills the K3-level at this stage of the study.*

### Introduction:

The introduction does not adequately address the state of the science. What are the benefits of dynamic feet compared to SACH, and why is this critical for LMICs? Reasons other than affordable technology such as a lack of prosthetists should be acknowledged as a part of the limited access to prostheses in LMICs. There exist highly affordable feet such as the Niagara foot, and this background into existing solutions is not adequately acknowledged. In fact, the Niagara foot is priced well below $100. Information about the manufacturing costs of feet needs to be dealt with and presented. What are the clinical and user issues with SACH feet? These limitations need to be presented.

*The chapter has been modified according to the comments.*

*The Niagara foot is now mentioned in the introduction. Also, the new version of the manuscript shows that the foot was included in our study and previously referred to as P_K3_C_. However, the Niagara foot is not used by the ICRC for two reasons: (1) the profile height prevents patients with long stumps from using it, and (2) the maximum size of 26 to 27 prevents tall patients with long feet from using it. Moreover, the Niagara foot has a limited choice of cosmetic shells available. The Niagara foot used to be advertised at 35 USD on www.niagarafoot.com. This website is now offline. We purchased Niagara feet through www.ortho-innovativ.de; the cost was 175 EUR per foot (incl. cosmetic shell).*

### Methods:

The authors define and use data relating to pitch angles and GRFs in the assessment and design of the prototype foot. Please provide some justification and citations to support that these metrics are in fact important aspects of dynamic foot design. Relating to this, the authors need to very clearly define and if applicable quantify what constitutes the differences between different foot classifications (K1 vs K2 vs K3). Please more clearly define what distinguishes a K3 foot from a K2 and K1 foot in terms of the functional or mechanical properties.

*The K-levels are generally defined in terms of the type of activities that patients can perform, and we agree that there can be different interpretations of those levels. For the reference prosthesis used, the K-level was taken from their published classification. The French HAS (Haute autorité de santé) defines the test standard in its publication available here (*[*https://www.has-sante.fr/upload/docs/application/pdf/cepp-467-avispiedsrestitutiondenergie.pdf*](https://www.has-sante.fr/upload/docs/application/pdf/cepp-467-avispiedsrestitutiondenergie.pdf)*). The definition adopted on this standard is based on the measured energy restitution for two loading cases: heel loading at 15° and forefoot loading at -20° measured in initial conditions, after 666'666, 1'333'333 cycles, and 2 million cycles. This point has been clarified in the manuscript in section 4.3*

The FEA lacks detail about constraints, loading, meshing, convergences etc. The optimization on line 225+ is vaguely described, and information is needed about what was actually done.

*Indeed, due to manuscript length restrictions, we reduced the details about these parameters. We have added information in the manuscript.*

*Mesh:
The ankle, the foam, and the cosmetic were meshed using quadratic solid tetrahedron elements (C3D10). The blade, represented by a composite shell with a variable thickness, was meshed using a quadratic shell element (S8R). The final FE characteristic element size was determined using a mesh convergence study on the load-displacement response. The overall characteristic element size is about 5mm for most parts of the prosthesis.*

*Constraint and Loading case:
The assembly was first oriented to model the desired loading angle and then clamped through a reference point (Rp) coupled to the prosthesis interface's upper horizontal surface. The vertical displacement of a rigid horizontal plate representing the ground was simulated as in the experimental setup test. Frictionless hard contact is used to model the foot-ground interaction.*

Line 292 – what is the 2% threshold based on?

*Based on our observations of the questionnaires, a difference lower than 2% on the hand-written scores seems too small to be significant, as the scores were sometimes imprecisely marked with a pencil. We obtained the 2% threshold empirically by dividing the width of the pencil marks by the width of the scale.*

*We have added a short explanation of this process in the manuscript.*

### Results:

The removal of 14 of 44 days is a significant amount (1/3 of data) and this could bias the results. How did the authors know that data were manipulated?

*The participants were responsible to start and stop the foot-worn sensors. Although they were instructed how to do so when the visited the rehabilitation center, some participants did not manage to correctly start the sensors, or they only did so for one leg. Without the data from the sound and prosthetic leg, we couldn't compare the results of the measurements.*

What is the basis for removing data related to extreme weather? Monsoons are a normal occurrence in Vietnam and should therefore not be excluded. More so, did the monsoon days effect one foot more than the other?

*Only two days were removed because of the heavy rainfalls. We did so because the participant in question was enabled to go to work due of the overflooded roads. Since his normal activity was affect by the weather and potentially not by the prosthetic foot, we decided to remove these measurements from the analysis as it could have biased the results. Also, the monsoon days removed where once for a patient equipped with a SACH foot, and once with P_PRO_.*

*We have updated the manuscript as such: … and extreme weather (i.e., heavy rainfalls during monsoon season* *affected the daily activity of two participants)*

Please provide some justification that bouts of walking are a good measure of prosthetic performance.

*We have added references to previous studies in section 3.6.2 of the manuscript. The number of walking bouts and the duration of these bouts are considered indicators of daily physical activity (of primary interest in rehabilitation medicine).*

The differences in bouts could be attributed to other factors. People in places such as Vietnam use walking as a primary means of mobility. They need mobility to pursue ADLs and other necessary tasks, so it seems unlikely that they could elect to walk more or less in light of these necessities, especially considering that the SACH foot appeared to work quite well for them. Please provide an explanation or hypothesis for these found differences.

*First, the assumption that walking is used mainly as a means of mobility to pursue ADLs in Vietnam is arguable. Some participants in this study lived in Hanoï, while others lived in the more rural area, several hundred kilometers away from the city. For the residents of Hanoï, motorbikes seemed to be the preferred option for mid to long shifts, with some of the participants reporting going for recreational walks rather than walking for work-related activities (e.g., taxi driver). Secondly, some patients reported playing with their kids as an activity carried during the two-day measurements. We think that comfort was one of the factors increasing the duration and number of walking bouts. The heaviest patient specifically noted that he appreciated the stiffer forefoot, which helped him walk faster.*

*Nonetheless, we agree that the daily activity evaluation will need to be carried out over a period longer than two days to prove the clinical relevance of the differences observed. This limitation is now discussed in the manuscript.*

Were the questionnaire results statistically significant? Please provide stats. Are the differences clinically significant?

*None of the questionnaire questions showed statistically significant differences in the paired-sample t-test (equality of means of values) and the Kruskal–Wallis test (comparison of the mean* ranks*).*

*We have added the above information in the manuscript and provided the questionnaire answers as supplementary data (S4).*

### Discussion and conclusions:

Need to explain the novelty and contributions of this work more clearly.

*We updated the discussion as such:*

*Thanks to its modular design, the tuning of its foam properties (i.e., density and compaction point), and the tuning of the blade thickness, the foot proposed in this study can be adapted to a wide range of patients' sizes and weights. Moreover, the chosen materials and related processing routes allow for performances comparable to high-end commercial products together with the targeted low manufacturing costs.*

The cost aspect needs to be discussed in comparison to other existing foot technologies designed for

### Minor:

Line 37 – remove 'both' since referring to 3 items

*We have removed the word "both". We meant that gait tests were performed in-lab and in real-world conditions.*

Line 73 – spelling of 'toe' is incorrect

*Corrected, thank you.*

Line 92 – need to specify the models and manufacturers of feet

*We have added the description of the commercialized prosthetic feet in the manuscript after each of the abbreviated codes. Hence, we now have: P_SACH_ (CR-SACH, CR Equipements, Switzerland), P_K3_C_ (Model 2, Niagara, Canada), P_K3_E_ (1D35, Ottobock, Switzerland), and P_K4_ (Vari-Flex, Össur, Iceland).*

Line 385 – what is 11'142?

*The number 11142 was in the French format. It is now corrected.*

Caption of Figure 6 stating the 13 and 5 participants is confusing and it is not clear how this relates to the actual sample size of 11 participants.

*There were indeed 13 participants in this study. However, only 5 among the 13 participants have tested P_PRO_. Note that P_PRO_ was tested during the last iteration of lab tests, while the other feet were tested multiple times (so the participants would not know which foot was a prototype).*

*We corrected the typo in section 3.2.1 of the manuscript and have added a short explanation in the caption of Figure 6.*

In table 1, the numbers do not make sense. For example, for the pitch angle at 50%, both the P3C and PK4 have the same value (22), yet they are noted as being significantly different. That is not possible. Also, the table is confusing, as it is not clear whether results are better or worse than the PK4. The direction matters and should be clear from the table or other figure.

*As mentioned in section 3.2.3, we calculated a pairwise distribution difference between PK4 and the other feet using the non-parametric Kruskal-Wallis test. This test compares the ranks of the samples rather than their mean. We preferred this method to an ANOVA test because the distribution of the samples did not seem Gaussian. We have added a sentence in the caption of Figure 1 to remind the readers of the Kruskal-Wallis test.*

*The aim of Table 1 is not to indicate if one foot is better than another. In this study, the ranking of the feet is based on their K-Level. Since Pk4 has the highest K-Level, we decided to compare the behavior of the other feet with Pk4. The objective was to observe when and where differences occur to later optimize the mechanics of our prototype and replicate the tests in lab.*

# Reviewer #2:

This paper presents a multi-disciplinary approach for designing and testing an affordable passive prosthetic foot suitable for the developing world context. It aims to create an affordable high mobility dynamic prosthetic foot to replace the currently distributed low mobility solid-ankle cushioned heel prostheses. The authors outline the design framework starting with design requirements, set through stakeholder discussions and gait studies of able-bodied walkers. Following with the development of the prosthetic foot design through material testing, structural optimization, manufacturing analysis, and mechanical testing. Lastly, they validated the designed prototype through gait studies, additional mechanical testing and a field trial.

The work described here has a lot of significance to the field in terms of the design approach as well as the resulting device. It is definitely worthy of a publication. However the work requires major revisions since additional information would be required to: support all the claims (mainly on the expected level of performance of the prosthetic foot prototype), justify the design and methodology choices (Why were this set of structural optimization target chosen, compared to standard measures such as roll-over shape or push off work? Can a foot that exhibit a certain load displacement behavior be categorized as fulfilling the WHO d4602 or K3 level performance? How close do you need to get to the P_K4 prosthetic foot to fulfill your requirements), and discussions on the gait analysis results would benefit from being put in perspective with existing literature (whole body propulsion, loading rate and peak vertical loads at heel strike, limitations of prosthetic boots usage compared to people with amputation, center of pressure progression, ankle moment etc…). In addition, the title of the work could be misleading as it refers to 'user-centric' approach but throughout the design process nor for the design requirements (given by the ICRC and WHO guidelines) were people with amputation consulted nor included in the development. People with amputation were included after the design of the prosthesis was completed. Lastly, minor revisions regarding the spelling, grammar, and phrasing issues would improve on the clarity of the work.

*We have updated the changed the title of the paper from "user-centric approach" to "functional approach".*

### Abstract:

Line 42: What specific aspects about the prosthetic foot performance was improved? By how much?

*We meant the performance of the patients, not prosthetic foot. So, we have updated the sentence as such: Additional tests were performed in the field with 11 trans-tibial amputees, who were more active with the proposed foot than with a SACH foot.*

### Introduction:

The authors should specify the level of performance of the current ICRC SACH foot to put in contrast with the target foot's performance. What specifically about the current ICRC SACH foot prevents it to meet the listed requirements?

*We have added a more detailed description of the limitations of the current prosthetic foot available for low- and middle-income countries (LMICs).*

Line 54: How about the Niagara foot, which meets the cost requirement, through bolt attachement requirement as well as the K2-K3 level activity? (Ziolo and Bryant 2002, Wellens 2011).

*The Niagara foot is now mentioned in the introduction. Also, the new version of the manuscript shows that the foot was included in our study and previously referred to as P_K3_C_. However, the Niagara foot is not used by the ICRC for two reasons: (1) the profile height prevents patients with long stumps from using it, and (2) the maximum size of 26 to 27 prevents tall patients with long feet from using it. Moreover, the Niagara foot has a limited choice of cosmetic shells available. The Niagara foot used to be advertised at 35 USD on www.niagarafoot.com. This website is now offline. We purchased Niagara feet through www.ortho-innovativ.de; the cost was 175 EUR per foot (incl. cosmetic shell).*

### Methods

#### Project Framework:

Line 94: The selected commercial prosthetic feet models should be specified to allow for reproducibility of the experiments, instead of abbreviated codes (P_K3_C).

*We have added the description of the commercialized prosthetic feet in the manuscript after each of the abbreviated codes. Hence, we now have: P_SACH_ (CR-SACH, CR Equipements, Switzerland), P_K3_C_ (Model 2, Niagara, Canada), P_K3_E_ (1D35, Ottobock, Switzerland), and P_K4_ (Vari-Flex, Össur, Iceland).*

Line 101: Which part of the in-lab biomechanical and mechanical evaluations where used to improve the model?

*The design optimization was mostly based on two key experimental data sets:*

- *The measured gait loads vs pitch angle for references dynamic foot prostheses*
- *The load-deflection and reaction moment v.s. load characteristics of reference prostheses (as well as intermediate physical prototypes of the developed design).*

*These clarifications were added to the manuscript.*

#### In-lab characterization of commercial prostheses:

Line 116: How long was the habituation period? Was all the tests for each participant with the commercial feet conducted on the same day?

*The habituation period was a minimum of 15 min long, but participants were free to ask for a more extended period. The order of the prosthetic feet was randomized for each participant. We have updated the document accordingly.*

Line 141: How were the shank, fore and rear foot frames defined? I assume the rear foot frame was defined using the three markers at the heel, the forefoot frames using the three markers at the forefoot and the shank frame, the four markers at the prosthetic ankle?

*Indeed, this is how the frames were defined. We removed the details about frame reconstruction before publishing to match the number of words allowed.*

Line 145: Why were the pitch angle and flexion angle considered instead of the more traditional ankle angle or shank angle?

*The goal of these tests was to observe when and where the feet differ with the prosthesis having the highest K-Level (Pk4), and then reproduce these tests in a more controlled environment using compression tests. In addition, the feet were different in their configuration. They differ in height and in encapsulation, making the calculation of the ankle angle more complicated. Hence, we preferred the pitch and flexion angles as these could be reproduced during the compression tests.*

Line 151: Why were the stance phase behavior at 30%, 50% and 100% of BW specifically chosen instead of others gait events/frames?

*We choose the loading of the feet for the same reasons then the previous point. The objective was to later reproduce the gait analysis tests in a more controlled environment with a compression test. Hence, we decided to look at specific loadings time-points of the stance phase. We selected +/- 30%, 50% and 100% empirically (the complete vGRF could be approximated from these six points).*

Line 158: the x-axis of Fig 3b seems mislabeled.

*We have not found an error in the label of Fig 3b.*

#### Design, materials and simulation:

Line 165: was the shell included in the optimization/modelling of the prosthesis?

*The optimization was performed in two steps: In the first phase, only the blade, ankle, and the higher density foam blocks used in the overload protection system (between blade and ankle at the heel, and between the contact surfaces of the overload protection system integrated into the top of the ankle). In a second phase, the design was refined using a full model, including encapsulating foam and shell. This second set of models was much more expensive and complex to run due to the foam's highly non-linear response. Ogen Hyper foam model for the encapsulation foam has a very low initial shear stiffness but becomes very stiff after compaction point. This behavior, combined with large deformation and contact, makes those simulations challenging. Hence the use of those models only in the last optimization phase.*

Line 168: How were the target performance outlined in d4602 translated into specific design requirements as the d4602 only outlines general walking aspects.

*The dynamic walking aspects involve the capacities to reach at least a certain level of deformation (or stored energy) at specific load cases such as heel strike and forefoot propulsion. Indeed, for example a dynamic foot must respond to heel strike in a sufficiently soft manner (but accept potentially large overloading) while being able to restore the deformation energy in the propulsion phase. In this work, the main targets for bio-mechanical response of the prosthesis were derived from the analysis of the gait loads vs pitch angle and static load-deflection response of reference dynamic foot prostheses for different pitch angles. Combining those two sets of data, we could infer a target prosthesis deflection for each pitch angle (under corresponding gait load). This criterion corresponds also roughly in imposing a certain level of stored energy (it would be the same if the system was linear). The design had to meet these requirements by fine tuning 2 parameters, the overall geometry of each part and their interfaces (to ensure the targeted stiffness and enough strength). A more detailed explanation*

Line 170: The term 'best gait pattern' should be clarified. Which aspects of the gait pattern were targeted? Does this term refer as getting as close as the K4 foot? How close?

*Yes, indeed, the target was to reach a prosthesis as close as possible to the reference K4 foot, but due to material/cost constraints and the requirements for a full encapsulation, we could not reach a 100% similar response in the end. The process is described in a more detailed manner in section 3.3.3 in the revised manuscript*

Line 182-183: the blade's elasticity and strength were optimized but what were the optimization targets? Was the blade optimized independently of the foam and ankle part?

*The blade layup was optimized experimentally through fatigue testing in bending in order to achieve the required fatigue strength in bending as well as reaching a suitable stiffness. The targets for the material optimization were set based on the FE analysis of the prosthesis (simulation of gait conditions to compute the max bending moments; design optimization by FE to determine the ideal bending stiffness to reach the target deflections under gait loads). Then the optimal blade composite layup properties were introduced in the model and the rest of the design parameters were optimized (ankle geometry, foam density and shape for the overload protection regions, blade thickness) to satisfy the biomechanics target response (load- deflection & moments at key gait phases, pitch angles) and strength requirements.*

Line 184: How much eversion/inversion was set as the target?

*The target eversion/inversion was ±15° and it was evaluated by ensuring that both sides of the foot were in contact when a neutral position load of 800N was applied. The load-deflection response of the prototype when contacting a 15° side slope was also compared to that of other reference prostheses such as Pk4 and showed a similar response (similar stiffness profile and same load at full contact closure, around 300 N). The target eversion/inversion angle has been added to the manuscript.*

Line 188: It is mentioned here that the foam density was used to accommodate different user weights, but only one foam density is later presented? Was the designed varied for user weights?

*It is not reported here for the sake of space, but the same design process has been repeated for foot sizes 22 to 28 with between two to three weight classes per size. The ankle design was kept identical between weight classes, and only the blade and density of the foam were adjusted. Those two parameters were found to be sufficient to satisfy the stiffness and strength criteria for different weight classes.*

*The foam density could be adjusted easily by changing the amount of foaming agent. In the present case, only one density was selected and was adapted to the one target foot size/weight we investigated in the project, which was on an average 0.45 g/cm^3^.*

'Different materials' (line 193) and 'specimens' (line 199) should include the details of the material that were tested/part of the selection process.

*Please refer to the supplementary information document for more info.*

Line 220: Why was a quasi-static FE model chosen instead of a dynamic FE model that would represent the prosthetic foot loading cycle and loading rate of the ISO tests?

*First, we did not have a setup for a dynamic test like ISO10328, and so we would not have been able to close the loop of optimization and iterate between experimental and modeling quickly. Second, dynamic simulations with highly non-linear foam models, large strain, and contact would have been nearly impossible to solve due to multiple sources of numerical instabilities (element distortion, conditional stability of the hyper-elastic foam model, highly non-linear stiffness change of the foam when reaching compaction point). We did try to perform those simulations but found it impractical in terms of stability and solution time. So, it was decided to evaluate the response at key times of the stance (static "snapshots" corresponding to several pitch angles). Except for rate effects, these static simulations would lead to similar simulated deflection and stresses than a dynamic model (acceleration terms are overall negligible, and the model is non-linear elastic with no contact friction, thus its response is path/history independent except for rate effects). We verified, however, experimentally, that the prosthesis's hysteretic response when loaded at 1Hz, 800N at a constant pitch angle was sufficiently low (~10% energy dissipation in the forefoot) and thus did not change the apparent stiffness of the prosthesis significantly. Thus, loading rate should not significantly affect the gait. It should also be noted that the foams used have been selected in order to be mostly elastic with minimal internal dissipation to maximize energy restitution.*

Line 224-228: Why was only the compression test of the selected commercial feet chosen as target for the optimization instead of the gait cycle tests?

*The compression tests were more controllable with better accuracy than the gait cycle. Moreover, we monitored more data during static testing, such as load-displacement and moment (used to calculate the center of pressure), and hysteresis. Finally, the measured load-deflection profiles can be interpreted to understand how the prosthesis works mechanically to externally applied forces and show important features such as a sudden change of stiffness at critical load/deflection points.*

Furthermore, the gait cycle tests included walking activities such as stairs, ramps and side to side stepping. Were these represented too in the compression tests?

*The contact with a sideways slope was tested to evaluate inversion/eversion (tests performed at 1/-15°). Forward / backward flexion (dorsi/plantar flexion) was represented in testing by considering pitch angles between 20° and -30° (20° dorsiflexion, -30° plantar flexions). Finally, the gait measurements gave us the ground reaction forces corresponding to each pitch angle during the gait cycle. This information was combined to interpret the compression tests at the correct gait load to determine the effective deflection for each pitch angle/stance during the gait.*

Was the objective equally weighting the deformation, reaction force and moment? Is the reaction force prescribed along with the plate orientation or was it an objective?

*The design optimization was mostly carried out based on trying to match the load-displacement response of the prototype to that of other reference dynamic foot prostheses for pitch angles from 20° to -30° with loads between 50N up to the corresponding gait load (ground reaction force measured at that pitch angle). The optimization was performed manually because of the need to adapt many geometry parameters in the CAD model, so we did not derive a weighted multi-objective criterion. Moreover, the strength constraints were usually limiting factors that would provide the maximum deflection, so the optimization work mostly consisted of removing as much as possible those strength constraints to reach a sufficiently high deflection (and thus energy storage) during a gate. In the end, the reaction moment response was considered in the last loop to fine-tune the shape of the blade and ensure a smooth motion of the center of pressure during gait.*

#### Mechanical compression tests

Should this section be presented before the design sections since these results were used as target during the design process?

*Thanks for the suggestion. It is difficult to move as this section defines how the target performance (input) was set and how the prototype was evaluated (output). For this reason, we decided to leave this section there.*

Line 241:’Feet hysteresis were quantified and evaluated' should these also appear in the results section?

*We can add new curves Annexed*

Line 245: How were the different pitch angles selected?

*The range from 20° to -30° was set to cover most of the gait cycles observed in-lab for the ground reaction force vs. pitch values. The particular pitch angles used to perform the optimization process were chosen in the first stage as 10° (high heel loading), 0° (neutral), 20° (high load in propulsion phase) to speed up the design process and then were extended to all other angles in the list for the final design optimization phase.*

#### Field Testing

Were these subjects given the same foot designed for an 80kg user as stated in the design section?

*Yes, they were. Due to time constraints and manufacturing costs, we were not able to produce feet of the different sizes and different stiffness.*

A copy of the adapted PEQ should be provided as supplemental information.

*It has been added to the supplemental information.*

Line 292: How was the threshold of 2% selected? Is that related to a statistical analysis?

*Based on our observations of the questionnaires, a difference lower than 2% on the hand-written scores seems too small to be significant, as the scores were sometimes imprecisely marked with a pencil. We obtained the 2% threshold empirically by dividing the width of the pencil marks by the width of the scale.*

*We have added a short explanation of this process in the manuscript.*

### Results

#### Prototype design

Line 319: What was the result of the optimization, how close did the prototype design get to the target objectives?

*The results can be evaluated by looking at Fig 8c and are discussed in section 3.4. In terms of mechanical response, we could reach, in most cases, deflection levels (and thus energy storage levels) between Pk3 and Pk4 prosthesis. The only notable exception was for high pitch angles (last propulsion phase), for which the prototype's elasticity fell below target despite model predictions being within an acceptable range of the objective. This stiffer behavior was observed only after producing a fully encapsulated prototype and is thus attributed to a stiffer response of the real encapsulation compared to the lab-produced samples. The text has been slightly modified to compare better the prototype's achieved mechanical performance in section 3.4.*

Line 343: How were the stress level/fatigue resistance values set?

*The target fatigue resistance values were set based on the finite element simulation of an intermediate design. The maximum bending moments & stresses in the blades and maximum von Mises stress in the ankle were calculated for different stance phases and used to set the material selection objectives in terms of strength.*

Line 363: Figure 8a caption refers to P_SACH but the graph represents P_PRO.

*This was a mistake. The caption has been changed.*

It is unclear in Fig 8.b what test was conducted? Was the vertical displacement the same for all of the prostheses and the corresponding load was measured?

*The opposite, the load was the same for each prosthesis, and the corresponding displacement was measured.*

The methods section only refers to loading cases being prescribed and not displacements. Was it from the biomechanical tests?

Figure 8 and 9: Why was only one P_K3 represented in these tests? Which one is it?

*For the sake of clarity, only one was represented: P_K3c. We edited the caption to clarify this point.*

### Discussion

Line 453: the P_PRO exhibit a more progressive motion of the center of pressure compared to the P_SACH but more abrupt compared to the other commercial feet? Does that allow the P_PRO to meet this requirement as there is a more abrupt change compared to the 3 higher level commercial feet?

*Only field-test, and human feedback would validate if the center of pressure's motion is progressive enough. It should be noted that the movement of the center of pressure of P_PRO_ is very similar in shape to P_K3_, but the center of pressure is usually 10-15 mm ahead in P_PRO_. The tuning of the patient's prosthesis could be altered slightly to compensate for a part of this offset. Compared to P_K4_, P_PRO_ does not have an as smooth transition. This is mostly because the P_PRO_ is a low-profile prosthesis with a split tow*, limiting the bending length of both the ankle and blade (shorter to leave space for the split tow).*

*(*for practical reasons, like wearing flip-flops, which is very common in many countries where humanitarian action takes place).*

*First feedback tells us that the prosthesis is considered stable by test subjects and that walking speed and other monitoring of activities (at least in the lab) give comparable results to P_K3_. Some reports of more limited front stability have been reported, but we do not have any statistically meaningful data published yet. As the project's main goal was to propose a more dynamic prosthetic food for humanitarian applications as a potential replacement for P_SACH_, we decided to highlight this demonstrable improvement mostly.*

*The discussion in section 5 addresses some aspects of the reviewer's question.*

Line 456: What explains the higher energy restitution after fatigue compared to the new state and how does it compare to the ther prosthetic feet?

*The behavior of the encapsulation foam evolves in the first few thousand cycles (< 10'000). As produced, it is stiffer and become more compliant after a short cycling phase. The foam then remains stable for the rest of the 2M cycles that were tested. This is thus due to a foam preconditioning effect. No significant change of stiffness during fatigue was observed on preliminary tests in which the prototype was not encapsulated.*

Was this test conducted with the foam and cosmetic shell?

*It was performed in the final configuration with a cosmetic shell.*

Line 471: Does the difference between the P_SACH and P_PRO in terms of overall satisfaction significant?

*No, it was not. We have added a sentence about this matter in the manuscript.*

Does the ankle moment, loading and displacement of the P_PRO enables the device to fulfill the k3 level requirement?

*The assessment of the K3-Level requires additional testing by a certified institution. We cannot assert that our foot fulfills the K3-level at this stage of the study.*

What were the limitations of the study? Did the prosthetic boot gait study match the walking pattern of prosthetic users?

*We have added the following paragraph about the limitation of the study in the conclusions:*

*Despite the promising results obtained, some limitations must be considered for the next steps of the project. First, the number of patients who took part in the field tests: 11 patients is too low for clinical evidence to be claimed at this stage; hence further testing at a larger scale is to be performed to strengthen the first findings. Second, even if we tested the prototype as close as possible to the ISO 10328 guidelines, complete testing by an independent certified laboratory is required to prove that the foot meets the standard.*
