# Supplementary material for: A functional approach towards the design, development, and test of an affordable dynamic prosthetic foot
Source: PLoS One. 2022 May 6;17(5):e0266656. doi: 10.1371/journal.pone.0266656 (PMC9075626; doi:10.1371/journal.pone.0266656)
Supplement: Attachment — Submitted filename: Reviewers_comments_main.docx [file pone.0266656.s008.docx]

**Reviewer #1:**

First, I would like to acknowledge the importance of the work aimed at addressing a challenging global problem. Secondly, I appreciate the multifaceted nature of the approach, considering a range of aspects relevant to solving the problem. The authors present analyses related to biomechanical, structural, finite element, cost, and various aspects of the design, as well as clinical field-testing results. The main challenge with this paper, is that it tries to present all these findings, and as is evident in many of the responses from the first review, there just is not enough space to treat each of these aspects with the level of detail and rigor that (in my opinion) is needed for a research paper. As such, there continue to be too many gaps in the paper, with important information missing. Unfortunately, the manuscript reads more like a student thesis project, than a research paper that one would expect to find in PLOS.

**Introduction**:

“According to the World Health Organization (WHO), 80% of people with disabilities live in low- and
middle-income countries (LMICs) [1], and the number of lower-limb amputees is likely to globally increase due to several factors such as diabetes, accidents, conflicts, and congenital disabilities [2]. Moreover, only 5 to 15 percent of people with disabilities living in LMICs have access to rehabilitation services. Prosthetic feet (further described as "feet" for ease of reading) with advanced biomechanical features ..” This section jumps from disability to lower-limb amputees, back to disability, and then very specific info related to feet. I think the flow of the information needs to be improved. Perhaps the information on feet deserves its own paragraph(s).

We reworked that paragraph for an easier reading and a better flow of information.

Line 58 ‘usually made of expensive materials and..’ – please be specific and provide an example of the expensive materials in question

We have cited the example of autoclaved carbon prepregs which are widely used for this application.

Line 74 ‘material and production processes (e.g., 3D-printing) have shown promising’ – its not clear why 3D printing is mentioned here, since it does not appear to play a role in the project.

This sentence was removed.

Line 79 – Is this manufacturing or the final cost? Most prosthetic devices have high margins, so the manufacturing cost is usually a small fraction of the final cost. In other words, many feet exist that cost under $100 to make, but are sold for many times that. Noting that it is the final cost that is important to the client. Please clarify and provide more insight about these costs.

As it is written, the ICRC asked for a total manufacturing (production) cost of 100 USD. A patent belonging to the ICRC, which is actually pending, allows them to discuss the margin made by potential licensed manufacturers by knowing exactly the production cost. The goal is to apply a low, or maybe differentiated, margin to promote the access to this product to the greatest number of people living in LMICs. At this stage, the margin that will be applied remains unknown.

In addition to K level, why is the weight capacity not considered along with the other design criteria

Thank you for pointing that out. The foot is designed for 3 different weight ranges for sizes 24 to 28, and 1 to 2 for smaller sizes, these weight ranges matching ISO 10328 P levels 3 to 6 depending on the foot size. The text was amended in that sense

**Methods:**

3.2.1. – please provide information or references that speak to the validity of using this protocol (able-bodied participants using the brace) to assess foot gait biomechanics.

We amended the paper and have added the reference of similar work, where it was stated: “... it (the results) shows that orthotic devices were good alternatives for simulating amputee gait...”

Line 149 – ‘The data from these units were used to empirically confirm that the previously developed algorithms for sound leg gait analysis [16,17] were functioning on the prosthetic foot and could be used for field tests.’ – It’s not clear what this statement aims to communicate.

We used the foot-worn IMUs of the in-lab tests to verify that the event detection of previously developed algorithms was also valid with the prosthesis of this study. The same algorithms were later used in-field.

‘However, we used the median, and the interquartile range (IQR) as inter-steps statistics (i.e., calculated over all the steps regardless of the subject) as histograms and the quantile-quantile plots (Q-Q plots) suggested a non-Gaussian distribution’ – Why? Please justify.

It is a common practice to use the median and IQR instead of the mean and STD, respectively, when the distribution of the samples does not seems Gaussian.

Line 199 – ‘…minimal use of carbon fiber reinforced material to limit cost, geometrical constraints due to the’Please elaborate on the above. In particular what is the cost of carbon fibre compared to other materials? This seems like it would be important information if one is trying to optimize costs.

The target cost for the blade included materials and manufacturing costs which were limited to 35 USD as mentioned in Table 2. The cost of carbon fiber varies a lot depending on the performance criteria such as the stiffness and fatigue requirements. As an example a high performance carbon fiber part can easily cost 5 times more than a more generic glass fiber part just based on material costs. Additionally it also depends on finding industrial partners that could produce this part at this target cost eventually. In our case we could identify such materials and manufacturing partners that satisfy these requirements. Other suitable materials for the blade part could be glass fiber based composites but they suffer from stiffness requirements for this application and would require thicker laminates ultimately leading to a heavier foot.

Lin 216 – Here and in a number of places the authors rely fully on references that speak to a particular protocol. This is not sufficient as it requires the reader in most cases to seek information elsewhere. The manuscript should possess adequate information/details to stand alone.

Sorry about that. We have amended the text to give a brief overview of the main aspects of this cost assessment. We have provided a cost comparison in the supplementary information which shows these elements.

Line 251 ‘The stiffness of the prototype …overloading.’ Please provide details about the manual optimization. More information is needed about how the optimization was conducted and variables applied. How is injection moulding a design constraint?

We added details concerning the fact that it was an incremental optimization of thickness and rib position on the FEA model. Concerning the injection, a collaborative work have been done with our industrial partner to optimize the position of the injection nozzle and design the part in a way to minimise drop pressure during the injection. Also LFT composites are more difficult to inject than regular injection grade polymers, so strict rules in terms of minimum / maximum shell thickness and flow directions need to be observed (proprietary information of the manufacturer).

Line 303 ‘In addition, we used a foot assessment questionnaire inspired from the Prosthesis Evaluation Questionnaire (PEQ) [24] to evaluate..’ – The PEQ is a validated questionnaire and unfortunately, using questions from it is not ideal. This is a major limitation that should be clearly recognized.

Indeed, we have amended the document in the discussion (Line 585) as such: “Although the questionnaire was inspired by the PEQ and not the actual validated PEQ, which we agree is not ideal and constitutes a limitation of this study, ..:”

Line 319 – ‘The questionnaire was composed of 14 explicit…’ – which questionnaire is this?

We have added the questionnaire in the supplementary materials.

**Results:**

Line 329 – ‘This number varied between subjects as invalid steps..’ – it is typical to collect data and exclude invalid step during collection – hence extra steps can be collected to replace the mishits. Why was this not done?

The steps which the participants missed by force plate were repeated. However, in some cases, it was challenging to detect missed-step visually. Hence some steps had to be removed during post-processing based on the position of the markers. Moreover, some steps were also removed because of occlusion in the markers, making the motion capture unreliable. We have amended the document to mention these cases better.

Line 364 – ‘Thermoplastic-based materials and processes were considered to limit the cost
associated with the complex shape of the ankle’ Please explain what this means.

This point was amended in the text directly: “A similar shape produced by traditional thermoset composite processes did not fit our target costs. Additionally thermoplastic processes such as injection molding provided full automation and low cycle times leading to cost savings.” We screened several different possibilities and also it was important to find industrial partners that are capable of producing such parts at our target costs. We identified this hybrid choice of different materials for each part to fit our conditions.

Table 2 – please explain the cost index

Amended in the text as well “This value is a percentage of the target cost (100 USD)”

Table 3 – the numbers are confusing. For example, it seems that the blade failed at 149,451 cycles(?)

Sorry for the seemingly confusing numbers but yes the blade at higher stress of 766 MPa failed after 149, 451 cycles of fatigue. We amended the text slightly to help the reader by adding the term “NF*: Not failed under these conditions”

The data from the PEQ and 6MWT need to be presented and discussed even if the findings were not significant. The fact that the prototype foot did not perform better in the 6MWT needs to be discussed. What have other studies found with this test when comparing SACH to higher end feet?

We have amended the discussion chapter (Line 905), and added a reference to previous work.

**Discussion**:

Line 433 – ‘..the foot displayed promising results.’ – Such as?

We meant this in terms of mechanical performance compared to other comparable prostheses and user acceptance. We added “*such as mechanical performanc*e” (that was measured in the laboratory) “*and user acceptance”* (feedback from field tests) to the sentence.

Line 434 - ‘the foot proposed in this study can be adapted to a wide range of patients' sizes and weights.’ – it is unclear where in the findings this was demonstrated.

Indeed, the fact that all the parts have been designed for 5 differents size ( 22, 24, 25, 26, and 28) cumulated with the possibility of tuning the blade thickness and the foam density (to adapt for weight classes) have been added .

Line 454 – ‘This mechanical feature was only observed for PK4 and could explain the feeling of “fluidity” perceived by..’ – Please clarify what data/results demonstrate this perceived fluidity.

A description has been added to the manuscript as requested.

Line 484 – ‘for PPRO led to a moderate deformation because of a higher stiffness..’ deformation of what? ‘This difference was not perceived as we used a cushioned heel..’ – by what or who was it not perceived? Please refer to the relevant results/data

The text was amended to clarify those points. The term “deformation” is used here to represent the relative displacement between the anchor point of the prosthesis and the heel contact point as measured in the laboratory setup. The “perception” aspect refers to the subject evaluation during walking tests. As a reference, the effect of a cushioned heel was evaluated also in laboratory with a prosthesis with / without shoes as shown below. It can be noticed that with a shoe, the difference of the vertical deflection at 15° loading (heel) is comparatively small. Which explains why subjects did not notice this difference in gait tests.

The upper figure presents the result of a mechanical compression test with the prosthesis set to have a heel contact (+15deg). Our prototype has been tested first without shoes, then with the shoes used during the laboratory test (Pproto_shoe1) and finally with a cushioned running shoe (Pproto_shoe2).

Shoes 1 Adidas:

Shoes 2 On Running model Cloudcruiser Midnight / Mars (2018)

Line 489 – spelling of ‘two’

We meant « toe ». We have corrected the typo.

‘The pragmatic approach using cost and performance as coupled guidelines allowed the team to step
away from a full carbon epoxy composite design,..’ reducing the amount of carbon also seems to have reduced the performance compared to the K4 foot. So it in fact appears to be a compromise. These points should be discussed. A carbon ankle (c section) will no doubt play a role in the dynamic performance of the foot. So how much performance is lost be replacing it with an injection moulded component? It would be interesting to discuss the elastic properties in relation to typical carbon fibre ones.

Yes, indeed it was all about finding a compromise. The text has been modified to reflect that clearly. The compromise made with LFT injection molded part were mostly on the fatigue strength (~150 MPa vs 750 MPa for carbon composite prepreg manufacturing) as shown in table 3. The elastic deformation limit in fatigue of the LFT composite was indeed larger (~1.5% strain) than the carbon fiber laminate (~0.6%). So the ankle design in LFT was mostly constrained by allowable stress in fatigue which required to reinforce it and thus limited it flexibility. However producing a prepreg carbon fiber ankle would have cost more than 4 times the price of the LFT part.

‘PPRO received a better average evaluation for six of the seven most significant questions’ – please include these results in the results section.

These results are available in Table 4

**Reviewer #2:**

This paper presents a multi-disciplinary approach for designing and testing an affordable passive prosthetic foot suitable for the developing world context. It aims to create an affordable high mobility dynamic prosthetic foot to replace the currently distributed low mobility solid-ankle cushioned heel prostheses. The work described here has a lot of significance to the field in terms of the design approach as well as the resulting device. It is definitely worthy of a publication. The authors have improved and addressed some of the reviewer’s comments but minor revisions should still be completed before submission. Including additional quantifiable measures to interpret the results would improve on the rigor and clarity of the manuscript. Instead of mentioning that the model is a ‘good match’ with the experiments providing the error in displacement or moments would be beneficial. The supplementary documents should be listed/referred to throughout the manuscript when relevant instead of at the end of the manuscript only. Similarly, the load displacement hysteresis curves, questionnaire information or questionnaire results mentioned in the reviewers’ response to be present in the supplementary document were not found.

**Methods**

Line 187: the ‘best’ gait pattern is a subjective assessment, be more specific and accurate by saying for example that feet were designed to replicate the pitch angles at different instances of stance.

Corrected as proposed

Materials and process selection methodology: refer to supplementary material 2

We have added the text to refer to the supplementary information for further details.

Mechanical simulation: Line 261: typo, either ‘cosmesis’ or ‘cosmetic shell’

Corrected

Field Testing: refer to the supplemental information for the questionnaire and it was not found in the supporting information listed in section 8.

Indeed, sorry. We added the questionnaire in supplementary material and amended the text.

**Results**

Prototype testing: be more specific regarding which figure was collected during gait testing and which one during the mechanical testing. From the manuscript it seems that the (a) and (c) were collected on the mechanical test setup while (b) is data from the gait testing.

Details added for (a) and I rephrase the (c)

Line 404: replace ‘gait GRF’ by GRF measured during gait testing to improve clarity.

We have updated the manuscript as suggested.

Line 411: ‘good agreement’ should be quantified, model predicted the displacement within ..mm

Corrected

Line 420: ‘peak at swing phase’ but the plot shown in Fig 8.a says moment during stance phase.

We consider that the end of the swing phase and the beginning of the stand phase overlap.

Figure 9a: the title during forefoot simplified stance phase could be clarify to convey the portion of stance phase where the forefoot is loaded.

“From 0 to -30deg” has been added

I assume here too that the CoP was back calculated from the moment and loading values in the compression test?

Yes, a descriptive sentence has been added.

‘the classification of the … by measuring energy restitution as defined in..” can you list the prototype’s energy restitution value compared to the other devices?

The respective restitution values measured in our laboratory tests are now reported in the manuscript.

Field testing: were the activity monitoring and questionnaire results statistically significant? Please include this information in the result section. In addition, the supplementary data (S4) does not seem to include the questionnaire answers as stated

The questionnaire is now available in the supplemental information. Comments on the statistical differences have also been added in the discussion.

**Discussion**

Line 513: typo ‘toe’ instead of ‘tow’

We meant « toe ». We have corrected the typo.

How do you explain that the CoP for small pitch angles was further down the foot compared to CoP at larger pitch angles for P_SACH? It is counter-intuitive that the CoP progression would be this way in gait testing. Additional comments regarding the results would be helpful.

This behaviour is due to the particular construction of the SACH foot that does not exhibit a flexible ankle. Indeed, as there is no flexibility of the ankle, close to the 0° transition, the contact area moves very quickly to the front of the foot for >0° pitch angle. Due to the small downwards angle of the sole surface compared to the tibial axis, at 0° pitch the contact area is very large and extends further than the end of the solid hankle. This is due to the fact that the solid ankle act as a “flat” plate at small angles and thus is vey effective at constraining the deformation of the foam (wide pressure cone). Even the base of the toes carry some load at small angles. When pitch angle increases however, the contact pressure becomes more and more concentrated around the end of the ankle which acts as a fixed pivot point. The toes being very flexible compared to the ankle in the SACH, their contribution becomes negligible at high angles. This concentration of contact pressure around the end of the solid ankle, combined with the high flexibility in shear of the foam (not constrained in shear) leads to this apparent reduction of the CoP position.

You mentioned that the questionnaire could only be accurately measured up to 2% in the method section (line thickness) but 3 out of the 7 questions had differences of 2% were still considered as an improvement/better answer from the participants. Should the comment regarding the improved feedback/perception only be valid for Q4 or maybe Q6 depending on the statistical analysis?

In the methods, it is mentioned that only the differences greater or equal to 2% were considered; hence 2% is included. This 2% threshold was empirically set based on the width of the pencil marks and the width of the scale. We agree that a 2% difference is small considering the number of participants which was stated as a limit of our study.

Comments to the reviewers regarding the inversion/eversion testing conducted to validate that the designed prostheses indeed enabled this functionality should be mentioned in the manuscript.

Thanks for this suggestion. The text now includes a short explanation and final results of the inversion/eversion tests.

Some information about the cost model and cost breakdown included in the reviewer’s documents should be included in the discussion or results to show that indeed the $100 threshold was achieved.

The text was amended “This combination of design, materials and manufacturing choices for each part in the prosthetic foot allows to reach the target performance and manufacturing cost of less than a 100 USD “. We apologize that during the previous submission to the reviewers we made an error by submitting highly confidential supplementary information ( S2: material characterization) and we kindly ask to revoke those specific documents submitted previously marked confidential that includes a supplementary information word document(SI_confidential_agilis paper) and a detailed cost analysis excel sheet(Cost modelling prostheses). Due to this additional information submitted earlier we understand that the reviewers have many queries about why we have not mentioned more detailed information in our article. The only reason we could not express ourselves more freely is due to the confidential information that we need to protect to ensure the industrialization and wide outreach of this product to all the patients in vulnerable areas worldwide through the ICRC. A revised supplementary information document for the materials and cost is submitted in this version. We hope the reviewers will understand this sensitive situation and support us in this effort.
