# Supplementary material for: A functional approach towards the design, development, and test of an affordable dynamic prosthetic foot
Source: PLoS One. 2022 May 6;17(5):e0266656. doi: 10.1371/journal.pone.0266656 (PMC9075626; doi:10.1371/journal.pone.0266656)
Supplement: Attachment — Submitted filename: Reviewers_comments_main.docx [file pone.0266656.s009.docx]

**Reviewer #1:**

Line 30 change ‘actors’ to ‘organizations’, also would remove ‘to a growing number of people’. Or improve the flow of the sentence otherwise.

Dear rewiever, the use of the word “actors” is on purpose since it is widely known among the humanitarian field that include different type of organizations (e.g. the ICRC, the United Nations, etc.). You will find an example of this wording in the peer-reviewed journal “ the International Review of the Red Cross”: <https://international-review.icrc.org/reviews/irrc-no-865-humanitarian-actors>. Another example on the website of the OCHA: <https://asiadisasterguide.unocha.org/III-humanitarian-actors.html>.

The sentence was modified to improve the flow.

Lines 33-35 – a reference is needed to support the assertion that there is a need for more dynamic feet. Also, it is not the low-income setting that would benefit, but rather the individuals with amputations that live in those settings. Please rephrase

We try to not add references in the abstract as common good practice. The same assertion is in the Introduction chapter, together with a reference (line 63)

The sentence was modified according to your comment.

Line 35-36 – this sentence requires references, so that the reader can look up information about these other attempt

A relevant example was added according to your comment.

Lines 37-46 should be better organized. Perhaps chronological order. i.e. development of design requirements, design and fabrication of foot, then testing. Also, should information should be included what population testing was done on (gait analysis, field etc)

Thank you for pointing that out, the paragraph was reworked accordingly.

line 55 – should say ‘services’

Corrected as proposed

line 58 -replace ‘like’ with ‘such as’

Corrected as proposed

line 74 – replace ‘the’ with ‘the feet’. Remove extra period.

Corrected as proposed

Line 75 – ‘needs in biomechanics, durability, aspect, compatibility with the PP technology, and cost.’ This part of the sentence does not make sense. Please reword.

The sentence was reworded and simplified for a better understanding.

Line 81 – the word ‘and’ is missing before the last point.

Corrected as proposed

Line 82 -which P level in particular is relevant or being targeted here?

Sentence amended accordingly

Line 85 – replace ‘prevent’ with ‘decrease’

Corrected as proposed

Line89 ‘ remove word ‘generic’ or replace with ‘main’. Also, where does the testing come in?

Corrected as proposed

Line 101- rephrase to ‘To gain insight into the mechanical response of existing feet..’

Corrected as proposed

Line 112 – should say’..was started’

Corrected as proposed

Line 115 – please rephrase this since simulations themselves re not doing the interacting

Sentence rephrased accordingly

Line 117 – iterations of what? Design interactions?

Sentence rephrased accordingly

Line 162 – replace ‘over’ with ‘for’

Corrected as proposed

Line 165 – Consider a more objective approach for assessing the normality of the distribution e.g. Shapiro-Wilk test. Please perform the test and provide results in the paper.

We have run the Shapiro-Wilk parametric hypothesis test. However, because the paper is already at the limits of the accepted manuscript length, we propose to report the results in the current document, which is also publicly available. The table below shows the p-values obtained from Shapiro-Wilk test with an alpha level at 0.05. The p-values higher than 0.05 are highlighted in bold.

| **Parameters** | **vGRF [%BW]** | **P_SACH_ (N=205)** | **P_K3_E_ (N=195)** | **P_K3_C_ (N=193)** | **P_PRO_ (N=55)** | **P_K4_ (N=192)** |
| --- | --- | --- | --- | --- | --- | --- |
| Pitch angle [°] | 30%_loading_ | 0.024 | 0.000 | 0.000 | **0.247** | 0.001 |
|  | 50%_loading_ | 0.001 | 0.000 | 0.000 | **0.078** | 0.000 |
|  | 100%_loading_ | 0.001 | 0.000 | 0.012 | **0.886** | **0.412** |
|  | 100%_pushing_ | 0.000 | 0.000 | 0.000 | **0.070** | 0.000 |
|  | 50%_pushing_ | 0.000 | 0.000 | 0.000 | 0.001 | 0.000 |
|  | 30%_pushing_ | 0.000 | 0.000 | 0.000 | 0.002 | 0.000 |
| Flexion angle [°] | 30%_loading_ | 0.000 | 0.000 | 0.000 | 0.000 | 0.000 |
|  | 50%_loading_ | 0.000 | 0.000 | 0.000 | 0.000 | 0.000 |
|  | 100%_loading_ | 0.001 | 0.000 | 0.000 | 0.000 | 0.000 |
|  | 100%_pushing_ | 0.000 | 0.000 | 0.000 | 0.003 | 0.000 |
|  | 50%_pushing_ | 0.000 | 0.000 | 0.000 | 0.000 | 0.000 |
|  | 30%_pushing_ | 0.000 | 0.000 | 0.000 | 0.002 | 0.000 |
| Time [%stance] | 30%_loading_ | 0.000 | 0.000 | 0.000 | 0.000 | 0.000 |
|  | 50%_loading_ | 0.015 | 0.000 | 0.000 | 0.000 | 0.000 |
|  | 100%_loading_ | 0.000 | 0.000 | 0.000 | 0.000 | 0.000 |
|  | 100%_pushing_ | 0.000 | 0.000 | 0.000 | 0.000 | 0.000 |
|  | 50%_pushing_ | 0.000 | 0.000 | 0.000 | 0.002 | 0.000 |
|  | 30%_pushing_ | 0.000 | 0.000 | 0.000 | 0.000 | 0.000 |

Line 168 – Should ‘time’ be plural?

Corrected as proposed

Line 183 – structural integrity my be a more appropriate term than reliability.

Corrected as proposed

Line 213 – a word after patient is missing. Patient characteristics?

Corrected as proposed

Line 306- consider replacing the word ‘comfort’ with ‘performance’ to more accurately capture the breadth of the outcome measures used

Corrected as proposed

Line 307 – what is a ‘healthy’ amputee. Please be more specific about how they were screened.

We meant “non-amputee”. We have corrected in the text.

Line 312 – checking for what?

For the sake of clarity, we have update updated the manuscript as such: “verification of the proper fitting and alignment of the prosthesis”. The certified orthotist/prosthetist verified that the prosthesis was properly fitting the patient and that the alignment of the foot was appropriate.

Line 313 – it’s more commonly referred to as the 6 minute walk test.

Corrected as proposed

Line 315 – replace ‘inspired’ by ‘adapted’

Corrected as proposed

Line 316 – ‘the feeling of the users’ is awkward wording.

We simplified with “comfort of users”.

Line 328 – be specific about which questionnaire is being referred to.

We added the reference to the questionnaire in appendix

Line 335-36 – need to more specific about the source of the data (i.e. the gait analysis)

The method used to extract the gait parameters is described in section 3.2.1 to 3.2.3. Could you please be more specific about the missing information?

Line 359 – replace ‘aspect’ with ‘design’

Corrected as proposed

Line 430 – how were instances when participants manipulated the data determined?

The participants had to change the sensors at the end of the first 24 hours of recordings. Although they were trained at VIETCOT, some did not manage to place the sensors correctly on the prosthesis which resulted in artefact movements of the sensors relative to the foot. We removed these trials based on our observations when we collected the sensors after the two days of recordings.

Line 432 – ‘A total of 11142 walking bouts have..’ is this for all the participants?

Yes, all participants combined.

Line 451 – the word ‘performances’ should be singular.

Corrected as proposed

Line 451 –‘ Moreover, the chosen materials and related processing routes allow for performances comparable high-end commercial products..’ this is somewhat of a pointless statement. More useful is to state whether comparable performance was actually achieved. My personal opinion is that there is a compromise that needs to be clearly noted as the design does not perform to the same level of high-end devices.

The sentence was rephrased. We initially intended to state that, in regards to the mechanical performance, the foot prototype could perform as well as Pk3_E, and even a bit better considering the hysteresis, but within the cost requirement.

Line 454-5 – The point of the sentence is unclear. What does the transdisciplinary nature of the work have to do with it being reported here?

You are right this sentence was badly phrased and is now improved, the word “interdisciplinary” was substituted to “transdisciplinary”. The goal is to demonstrate that a multidisciplinary collaboration of laboratories having a limited knowledge on prosthetics evolved towards an interdisciplinary approach of the subject/challenge over the course of the study.

Line 467 – chance ‘inside’ to ‘within’

Corrected as proposed

Line 500- change to ‘during the heel…’

Corrected as proposed

Line 545- ‘prototype's conception objective’ is awkward phrasing

Sentence rephrased accordingly

Line 547 – change to ISO structural testing standards

Corrected as proposed

**Reviewer #2:**

This paper presents a multi-disciplinary approach for designing and testing an affordable passive prosthetic foot suitable for the developing world context. It aims to create an affordable high mobility dynamic prosthetic foot to replace the currently distributed low mobility solid-ankle cushioned heel prostheses. The work described here has a lot of significance to the field in terms of the design approach as well as the resulting device. It is definitely worthy of a publication.

The authors have edited the manuscript with improved the clarity, details, and addressed the reviewer’s comments but minor small revisions should be implemented before submission.

**Introduction**

Line 53: Typo ‘..’

Corrected as proposed

Line 62: Please explain ‘level 3 activities’ and the remaining mobility levels (Line 74) instead of just adding the reference as they might be confused with the Medicare K levels introduced later.

Corrected as proposed

Line 64: ‘ Due to its high profile’, what is considered a high profile and what was the prosthetic height target set for the author’s prosthetic feet?

The profile height was compared to a SACH foot as the reference (allowing less fitting restrictions du to stump length). The importance of profile height was emphasized again on lines 89-90.

**Methods**

Line 223: Typo ‘ this research, Scalability’

Corrected as proposed

**Results**

Table 3: Typo ‘ Carbon Fibre’

Corrected as proposed, also in Introduction.

**Discussion**

Line 513: “A closure force of less than 40% vGRF was found for PPRO which is comparable
513 to that of reference prostheses such as PK4.” What was the closure force of PK4 so that the reader can also deem it comparable?

Sentence amended with measured force value. Compression test curve added to supplemental material.

Questionnaire supplemental material: In the questionnaire questions, 14 items are listed but only 7 are reported in the results both in the supplemental material as well as in the manuscript. Why were the other 7 questions omitted?

As described in section 3.6.2: “… results reported only for the questions where we observed a difference greater or equal to 2% (threshold empirically set based on the width of the pencil marks and the width of the scale)”

The hysteresis curves have should been included in supplemental material as they are mentioned in the test methodology and discussions. In addition, are the energy restitution averaged across the different loading cases or only taken from a single loading case?

The sentence was amended accordingly, including the required details. Hysteresis curves were added to supplemental material.
